# Increased triacylglycerol production in oleaginous microalga *Neochloris oleoabundans* by overexpression of plastidial lysophosphatidic acid acyltransferase

**DOI:** 10.1186/s12934-019-1104-2

**Published:** 2019-03-13

**Authors:** Wipa Chungjatupornchai, Kanchanaporn Areerat, Sirirat Fa-Aroonsawat

**Affiliations:** Institute of Molecular Biosciences, Mahidol University, Salaya Campus, Phutthamonthon, Nakhon Pathom 73170 Thailand

**Keywords:** Biofuels, Lysophosphatidic acid acyltransferase (LPAAT), 1-Acyl-*sn*-glycero-3-phosphate acyltransferase (AGPAT), Microalgae, Lipids, Edible oils

## Abstract

**Background:**

Microalgae are promising sources of lipid triacylglycerol (TAG) for sustainable production of natural edible oils and biofuels. Nevertheless, products derived from microalgal TAG are not yet economically feasible; increasing TAG content via targeted genetic engineering of genes in TAG biosynthesis pathway are important to achieve economic viability. To increase TAG content, oleaginous microalga *Neochloris oleoabundans* was genetically engineered with the endogenous enzyme lysophosphatidic acid acyltransferase (NeoLPAAT1) responsible for plastidial TAG biosynthesis

**Results:**

NeoLPAAT1 was found to contain all canonical motifs attributed to LPAAT proteins, two hypothetical membrane-spanning domains and a putative chloroplast transit peptide, indicating as a member of plastidial LPAAT type 1 subfamily. The *NeoLPAAT1*-expression cassette integrated in *N. oleoabundans* transformant was confirmed by PCR. The neutral lipid content in the transformant detected by Nile red staining was 1.6-fold higher than in wild type. The *NeoLPAAT1* transcript was twofold higher in the transformant than wild type. Considerably higher lipid quantity was found in the transformant than wild type: total lipid content increased 1.8- to 1.9-fold up to 78.99 ± 1.75% dry cell weight (DCW) and total lipid productivity increased 1.8- to 2.4-fold up to 16.06 ± 2.68 mg/L/day; while TAG content increased 2.1- to 2.2-fold up to 55.40 ± 5.56% DCW and TAG productivity increased 1.9- to 2.8-fold up to 10.67 ± 2.37 mg/L/day. A slightly altered fatty acid composition was detected in the transformant compared to wild type; polyunsaturated fatty acid (C18:2) increased to 19% from 11%. *NeoLPAAT1-*overexpression stability was observed in the transformant continuously maintained in solid medium over 150 generations in a period of about 6 years.

**Conclusions:**

Our results demonstrate the considerably increased TAG content and productivity in *N. oleoabundans* by overexpression of plastidial *NeoLPAAT1* that are important for products derived from microalgal TAG to achieve economic viability. Plastidial LPAAT1 can be a candidate for target genetic manipulation to increase TAG content in other microalgal species with desired characteristics for production of natural edible oils and biofuels.

## Background

Microalgae are promising sources of lipid triacylglycerol (TAG) for sustainable production of natural edible oils as an alternative to plant derived oil [1, 2] and biofuels as an environmentally safe alternative to fossil fuels [3, 4]; because they possess short life cycles, perform photosynthesis, require non-arable land and absorb a large amount of CO_2_. Nevertheless, there are several challenges that need to be overcome before products derived from microalgal TAG can be economically produced at a commercial scale, one of which is the lack of microalgal strains with high TAG content [3, 5]. Increasing TAG content in microalgae could be achieved by targeted genetic engineering of genes in TAG biosynthesis pathway [4, 6, 7].

The TAG biosynthesis pathway in microalgae is not yet fully understood but considered to be most similar to that operating in higher plants [8]. In the model plant *Arabidopsis thaliana* and the model microalga *Chlamydomonas reinhardtii*, two sets of homologous enzymes catalyze two distinct and parallel TAG biosynthesis pathways, one in the plastid (prokaryotic pathway) and the other in the endoplasmic reticulum (ER; eukaryotic pathway) [9-13]. The ER pathway has been shown as an important route of TAG biosynthesis, however, increasing evidence suggests that the plastidial pathway also plays an important role in TAG biosynthesis in *C. reinhardtii* [13, 14]. In TAG biosynthesis pathway, lysophosphatidic acid acyltransferase (LPAAT or LPAT; EC 2.3.1.51) also known as 1-acyl-*sn*-glycero-3-phosphate acyltransferase (AGPAT) catalyzes the acylation of the *sn*-2 position of lysophosphatidic acid to generate a key intermediate, phosphatidic acid [15]. *C. reinhardtii* has been shown to possess CrLPAAT2 localized to ER membranes and CrLPAAT1 localized to plastid [11, 16]. Overexpression of *LPAAT1* for enhancing TAG accumulation has been attempted so far in very few microalgal species. *CrLPAAT1* overexpression in *C. reinhardtii* led to > 20% increase in oil content [11] and *LPAAT1* (*AGPAT1*) overexpression in diatom *Phaeodactylum tricornutum* led to increase in lipid content by 1.81-fold [17]. However, no *LPAAT1* overexpression has been reported so far in oleaginous microalga *Neochloris oleoabundans*.

*Neochloris oleoabundans,* a taxonomic synonym of *Ettlia oleoabundans* [18], is a promising source of TAG; because under nitrogen starvation condition, it produces lipids 36–54% of its cell dry weight and up to 80% of its total lipids is TAG [19]. However, the knowledge concerning *N. oleoabundans* is very limited; no genomic sequences are available. To enable targeted genetic manipulation of TAG biosynthesis, the stable nuclear transformation system of *N. oleoabundans* has been established [20] and the cDNA encoding LPAAT1 of *N. oleoabundans* (*NeoLPAAT1*) has been cloned [21].

In this study, we tested whether overexpression of endogenous plastidial *LPAAT1* would affect TAG biosynthesis in oleaginous microalga. The plastidial *LPAAT1* cDNA sequence of *N. oleoabundans* (*NeoLPAAT1*) was characterized. The *NeoLPAAT1*-overexpressing *N. oleoabundans* was generated and characterized with regards to growth and neutral lipid accumulation in the cells, lipid content and productivity, and fatty acid composition.

## Results

### Comparative homologue of NeoLPAAT1

**Fig. 1:**
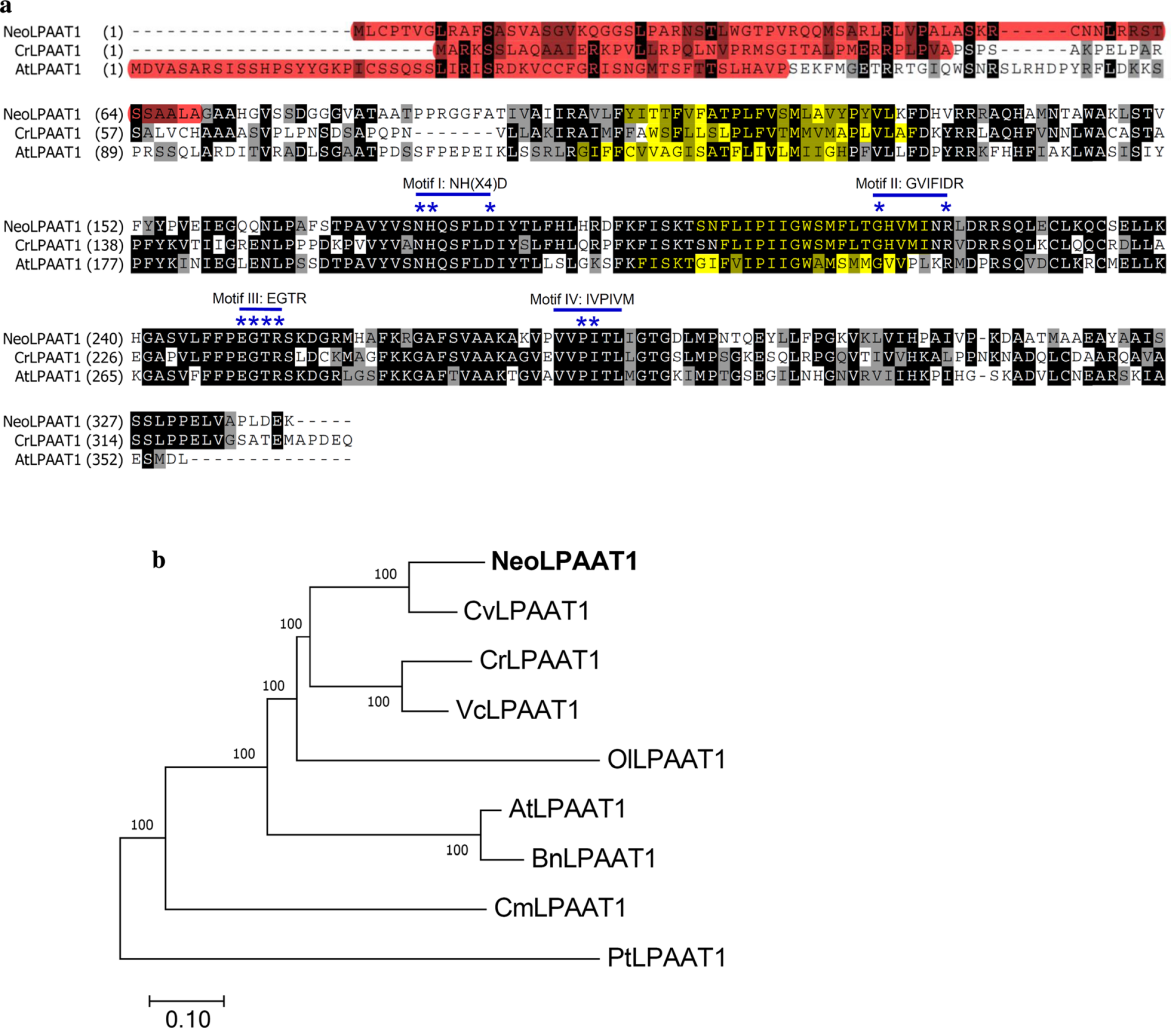
Comparison of NeoLPAAT1 with other plastidial LPAAT1. **a** Sequence alignment of CrLPAAT1, AtLPAAT1 (ATS2) and NeoLPAAT1. The amino acid sequences of CrLPAAT1 (*Chlamydomonas reinhardtii*, Uniprot: A8J0J0), AtLPAAT1 (*Arabidopsis thaliana*, Uniprot: Q8GXU8**)** and NeoLPAAT1 (*N. oleoabundans*, GenBank protein id: AUS8446) were aligned using ClustalW algorithm [22]. The chloroplast transit peptides of CrLPAAT1, AtLPAAT1 [15] and NeoLPAAT1 predicted using PredAlgo [25] are highlighted in red. Membrane-spanning domains predicted by TMHMM [24] are highlighted in yellow. Conserved and similar residues are indicated by black and gray boxes, respectively. The conserved motifs I, II, III and IV [23] are indicated. The highly conserved residues in the motif are indicated by asterisks. **b** Phylogenetic analysis of NeoLPAAT1. The phylogenetic tree was constructed using MEGA 7 [26]. The percentage of neighbor-joining boot strap replications is shown above each node. Scale bar indicates 0.1 amino acid substitutions per site. Green algae: NeoLPAAT1 (*N. oleoabundans*, GenBank protein id: AUS8446), CvLPAAT1 (*Chlorella variabilis*, Uniprot: E1ZQN6), CrLPAAT1 (*C. reinhardtii*, Uniprot: A8J0J0), VcLPAAT1 (*Volvox carteri*, Uniprot: D8U1V6) and OlLPAAT1 (*Ostreococcus lucimarinus*, Uniprot: A4S0H0). Red alga: CmLPAAT1 (*Cyanidioschyzon merolae*, Uniprot: M1V4N2). Diatom: PtLPAAT1 (*Phaeodactylum tricornutum*, Uniprot: B7FQL9). Plants: AtLPAAT1 (*A. thaliana*, Uniprot: Q8GXU8**)** and BnLPAAT1 (*Brassica napus*, Uniprot: Q9LLY4)

The *LPAAT* cDNA of *N. oleoabundans*, cloned previously (GenBank: MF706164; protein id: AUS8446) [21], was designated as *NeoLAPAAT1* in this study. When compared with the well characterized LPAAT1 of *Chlamydomonas reinhardtii* (CrLPAAT1) [11] and *Arabidopsis thaliana* (AtLPAAT1/AtATS2) [15] using ClustalW program [22], NeoLPAAT1 was found to share high level of amino acid sequence identity to CrLPAAT1 (46.8%) and AtLPAAT1 (37.3%) (Fig. 1a). All four canonical motifs attributed to LPAAT proteins [23]: motif I, NH(X4)D; motif II, GVIFIDR; motif III, EGTR and motif IV, IVPIVM were observed in NeoLPAAT1. Two hypothetical membrane-spanning domains as predicted by TMHMM v2.0 [24] were also detected in NeoLPAAT1 (highlighted with yellow in Fig. 1a). In addition, N-terminus of NeoLPAAT1, as predicted by PredAlgo [25], contained a putative chloroplast transit peptide ( ~ 69 amino acids, highlighted with red in Fig. 1a), suggesting that NeoLPAAT1 could be targeted to the chloroplast of *N. oleoabundans*. Therefore, the results indicated that NeoLPAAT1 is a homolog of the plastidial LPAAT1 of *C. reinhardtii* and *A. thaliana.* In the phylogenetic tree constructed using MEGA 7 [26], NeoLPAAT1 sharing 66.0% amino acid identity to *Chlorella variabilis* CvLPAAT1 was grouped in the same clade (Fig. 1b), suggesting that NeoLPAAT1 possessed the closest evolutionary relationship with CvLPAAT1.

### Selection of *N. oleoabundans *transformants

**Fig. 2:**
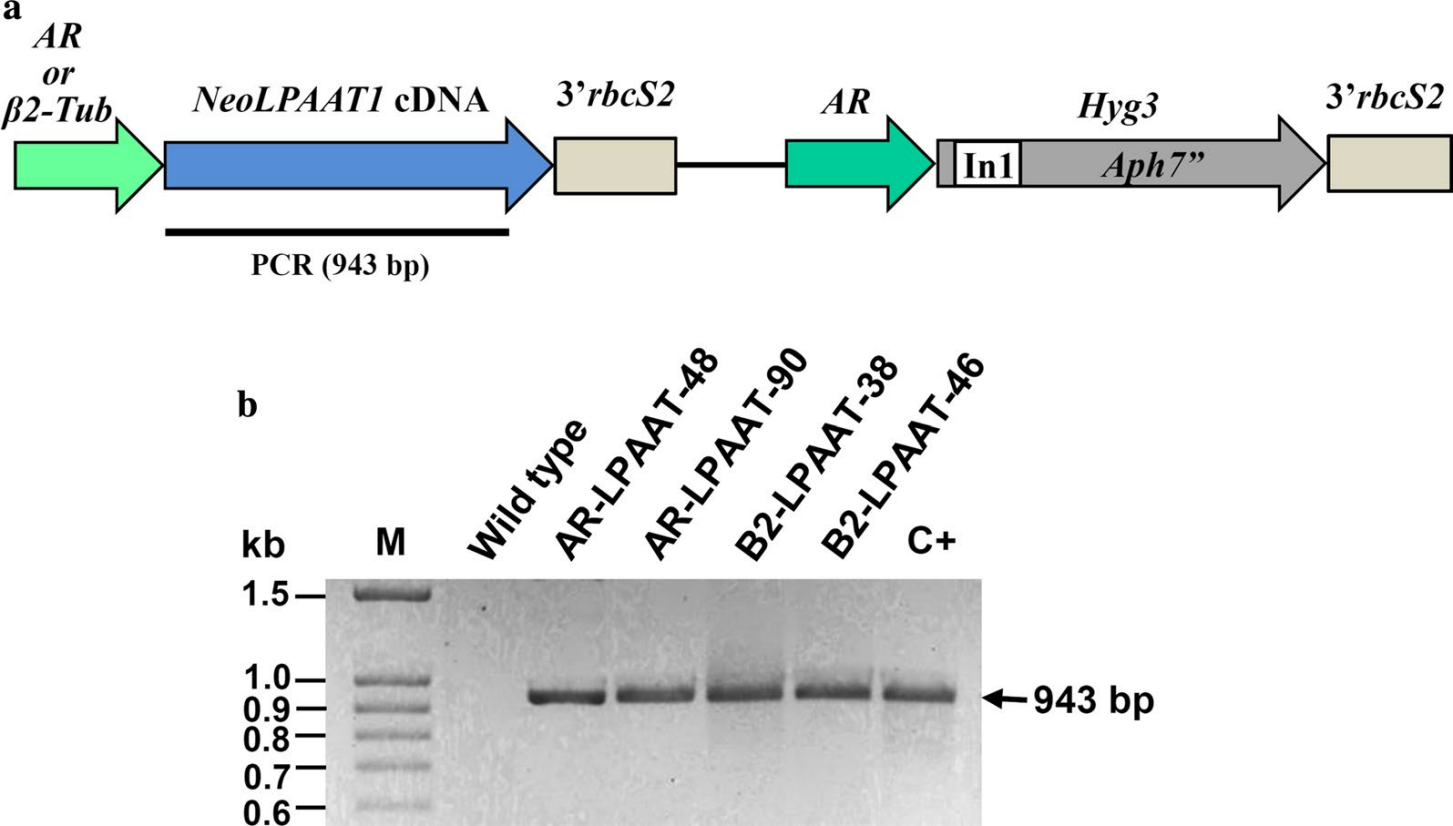
Generation of *N. oleoabundans* transformants. **a** Schematic map of plasmids pAR-LPAAT and pB2-LPAAT used for electroporation in *N. oleoabundans*. The *NeoLPAAT1* cDNA (GenBank accession no.: MF706164) [21] was expressed under the control of either promoter *AR* (*HSP70*-*RBCS2*) [44] or *β2-Tub* (*β2*-*tubulin*) [45] and harbored *3′rbcS2* [47] at 3′end. *Hyg3* gene, used as selectable marker [20, 46]. The 943-bp PCR amplicon, denoted by a line. **b** Genomic PCR detection of transformants harboring the *NeoLPAAT1*-expression cassettes. Genomic PCR of transformants AR-LPAAT, B2-LPAAT and wild type was performed with primers specifically bind to *NeoLPAAT1* coding sequence. The 943-bp amplicon was detected in the transformants but not in wild type (used as negative control). Lanes M, 100-bp DNA ladder; C+ , plasmid pAR-LPAAT (used as positive control)

*Neochloris oleoabundans* was electroporated with plasmids pAR-LPAAT and pB2-LPAAT containing *NeoLPAAT1* cDNA under the control of promoters *HSP70*-*RBCS2* (*AR*) and *β2*-*tubulin* (*β2*-*Tub*), respectively (Fig. 2a). These promoters have been shown to function in *N. oleoabundans* [20, 27]. The resulting transformants AR-LPAAT and B2-LPAAT were selected on BBM agar supplemented with hygromycin B. To screen for clones with potential high-neutral-lipid accumulation, about 100 colonies selected from each plasmid transformation were grown on BBM agar plates for 14 days and then stained with Nile red yielding intensely fluorescence in a neutral lipid environment [28]. Transformants AR-LPAAT-48, AR-LPAAT-90, B2-LPAAT-38 and B2-LPAAT-46 found to have high Nile red fluorescence intensity were selected for subsequent experiments.

### Detection of *NeoLPAAT1*‑expression cassette integration

The integration of *NeoLPAAT1-*expression cassettes in *N. oleoabundans* was analyzed using genomic PCR with primers specific to *NeoLPAAT1* coding sequence. The expected 943-bp amplicon was detected in the selected transformants: AR-LPAAT-48, AR-LPAAT-90, B2-LPAAT-38 and B2-LPAAT-46, but not in wild type (Fig. 2b). The 943-bp amplicon subjected to DNA sequencing was confirmed to be *NeoLPAAT1* coding sequence. Therefore, the *NeoLPAAT1*-expression cassettes were successfully introduced into the transformants. The amplicon from the resident *NeoLPAAT1* gene including introns was 1865 bp. Because one of the two primers used in this PCR was located on two exons, the resident *NeoLPAAT1* gene was not amplified. The PCR-positive transformants were further analyzed for growth characteristics.

### Growth of the transformants

**Fig. 3:**
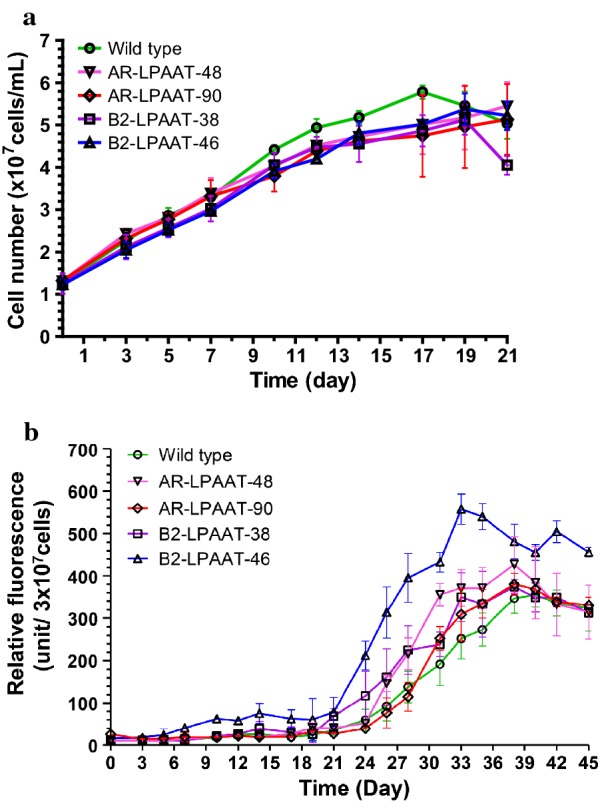
Growth and neutral lipid accumulation of transformants AR-LPAAT and B2-LPAAT. **a** Growth curve and **b** Neutral lipid accumulation of the transformants and wild type during +N growth condition. Neutral lipid accumulation was evaluated using Nile red staining. Transformant B2-LPAAT-46 was found to reach maximum Nile red fluorescence earlier and higher than wild type. Each value represents mean ± SD (n = 3)

To evaluate the effect of *NeoLPAAT1* overexpression on growth, we analyzed the growth curve under nitrogen-sufficient ( +N) condition of the transformants. All selected transformants showed overall similar growth compared to wild type, whereas slightly lower growth during the stationary phase (Fig. 3a). The doubling time during exponential growth of the transformants (4–5 days) was not significantly different from that of wild type (at *p* < 0.02). Therefore, *NeoLPAAT1* overexpression did not have an apparent effect on growth of the selected transformants AR-LPAAT-48, AR-LPAAT-90, B2-LPAAT-38 and B2-LPAAT-46.

### Neutral lipids of the transformants

To investigate the potential high-lipid production of the selected transformants, neutral lipids in the cells during +N growth condition were monitored by Nile red staining. The exponential growth of *N. oleoabundans* has been shown to accompany the decrease in N concentration; N-limited growth can stimulate the cells to produce more lipids [29]. The levels of neutral lipid accumulation in transformants AR-LPAAT-48, AR-LPAAT-90, B2-LPAAT-38 and B2-LPAAT-46 were different; they were higher than that in wild type (Fig. 3b). Whether the different levels of neutral lipid accumulation in the transformant clones are due to copy number or integration positional effect of the *NeoLPAAT1-*expression cassettes remains to be investigated. Among the transformants, B2-LPAAT-46 was found to reach maximum neutral lipid content (day 33) earlier than wild type (day 38) and have the highest neutral lipid content which increased to 1.6-fold compared to the maximum content in wild type (Fig. 3b). Therefore, transformant B2-LPAAT-46 was selected for subsequent experiments.

### Evaluation of *NeoLPAAT1 *transcript

**Fig. 4:**
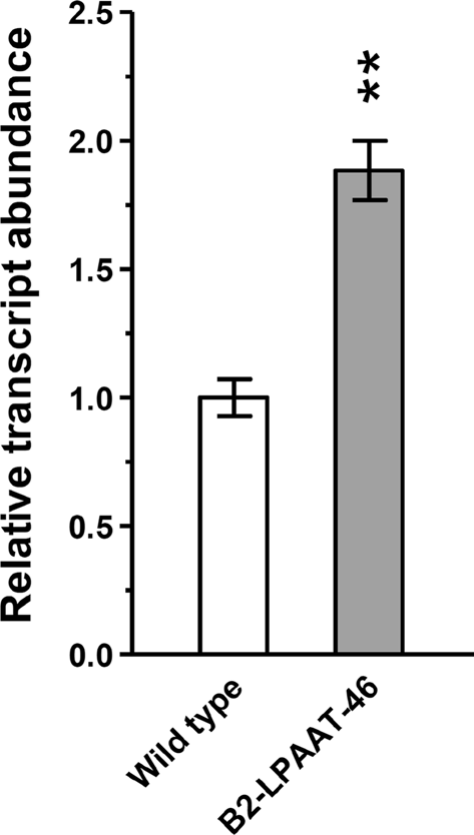
Relative *NeoLPAAT1* transcript abundance in transformant B2-LPAAT-46. The levels of *NeoLPAAT1* transcript under −N growth condition were determined by quantitative real-time PCR. The values are normalized to the expression level of endogenous *NeoActin.* Each value represents mean ± SD (n = 3). Significant difference between transformant B2-LPAAT-46 and wild type is indicated (**p < 0.01, *t* test)

The up-regulated LPAAT (AGPAT) transcripts in response to N-starvation has been observed by transcriptomic analysis of *N. oleoabundans* [30]. To evaluate whether the *NeoLPAAT1*-expression cassette integrated in the transformant expressed at transcriptional level, the relative *NeoLPAAT1* transcript abundance in the transformant B2-LPAAT-46 cultured under −N condition was determined by quantitative real-time PCR (qPCR) using *NeoActin* transcript as a reference. Transformant B2-LPAAT-46 was observed to have *NeoLPAAT1* transcript increased twofold compared to wild type (Fig. 4), indicating that the increased transcript was enhanced by overexpression of *NeoLPAAT1*.

### Lipid productivity analysis

**Fig. 5:**
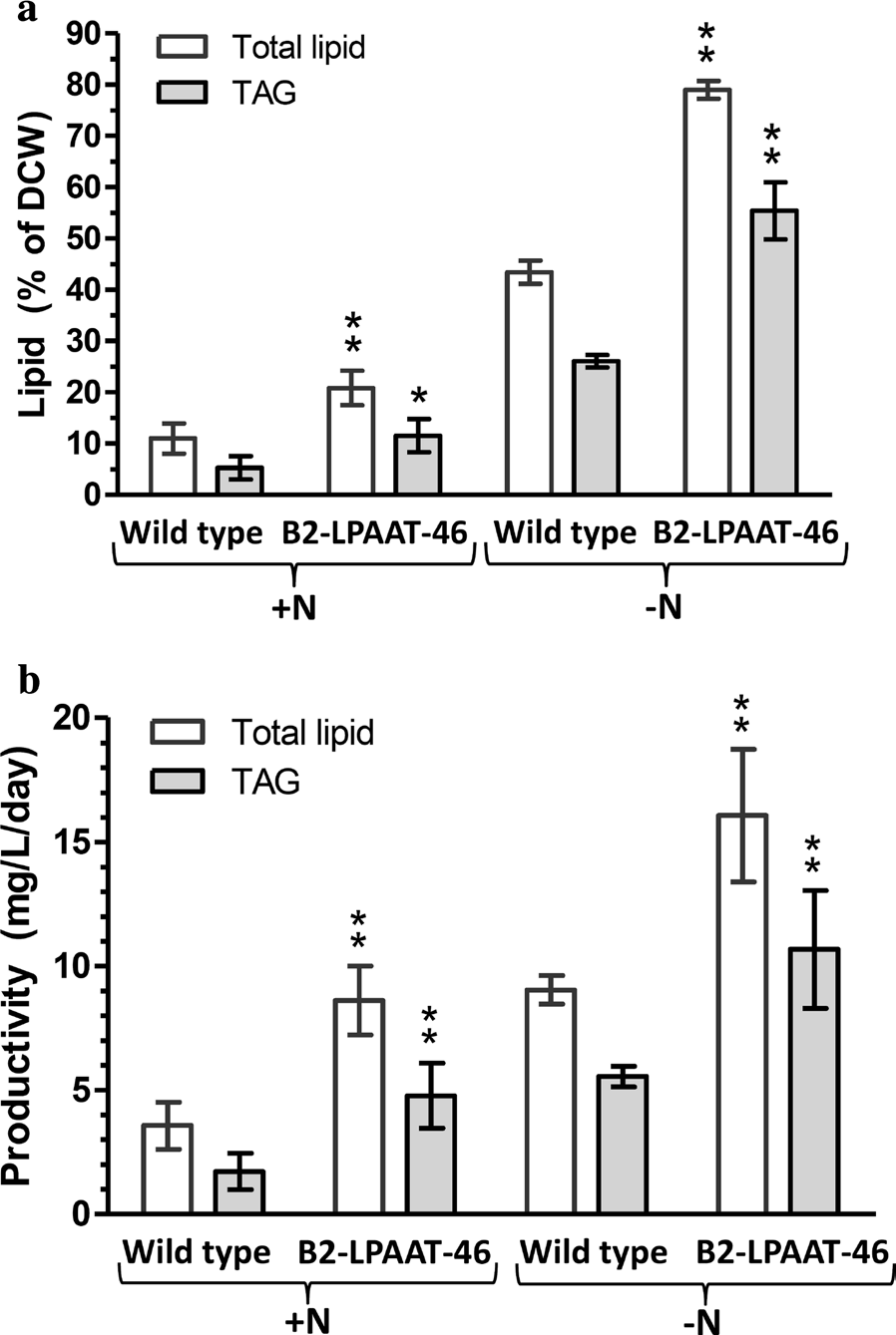
Lipid analysis of transformant B2-LPAAT-46. **a** Lipid content and **b** Lipid productivity. Lipids extracted from transformant B2-LPAAT-46 and wild type grown under +N and −N condition. Each value represents mean ± SD (n = 3). Significant difference between transformant B2-LPAAT-46 and wild type in the same growth condition is indicated (*p < 0.05, **p < 0.01, *t* test)

Transformant B2-LPAAT-46 and wild type were not significantly different in doubling time under + N growth condition, followed by N-limited growth that stimulated neutral-lipid accumulation at maximum level on day 33 and 38, respectively (Fig. 3a, b). *Neochloris oleoabundans*, like many microalgae, accumulates neutral lipids under N-starvation condition [19, 29, 31]. To accelerate the lipid production, transformant B2-LPAAT-46 and wild type were cultured under −N condition and the neutral-lipid accumulation as monitored by Nile red staining reached maximum level on about day 18 to 20. The dried cells were determined gravimetrically as dry cell weight (DCW). Biomass under −N condition was not significantly different between the transformant B2-LPAAT-46 (461 ± 65 mg DCW/L) and wild type (462 ± 27 mg DCW/L) (*p* < 0.05). Thus, the biomass was not affected by *NeoLPAAT1* overexpression. Lipid analysis was performed in transformant B2-LPAAT-46 and wild type cultured under +N and −N condition with the maximum neutral lipid accumulation monitored by Nile red staining. Because TAG is the major component for natural edible oil and biofuel production, the TAG content separated from total lipids using thin-layer chromatography (TLC) was quantified. Up to 70% of total lipids extracted from transformant B2-LPAAT-46 and wild type were TAG (Fig. 5a). The lipid content and productivity in transformant B2-LPAAT-46 were compared to wild type: under +N condition, total lipid content in transformant (20.8 ± 3.4% DCW) increased 1.9-fold, TAG content (11.55 ± 3.2% DCW) increased 2.2-fold, total lipid productivity (8.61 ± 1.39 mg/L/day) increased 2.4-fold, TAG productivity (4.77 ± 1.32 mg/L/day) increased 2.8-fold; under −N condition, total lipid content in transformant (78.99 ± 1.75% DCW) increased 1.8-fold, TAG content (55.40 ± 5.56% DCW) increased 2.1-fold, total lipid productivity (16.06 ± 2.68 mg/L/day) increased 1.8-fold, TAG productivity (10.67 ± 2.37 mg/L/day) increased 1.9-fold (Fig. 5a, b). Therefore, transformant B2-LPAAT-46 was observed to considerably increase in lipid accumulation when compared to wild type: the total lipid content and productivity increased 1.8- to 1.9-fold and 1.8- to 2.4-fold, respectively; while the TAG content and productivity increased 2.1- to 2.2-fold and 1.9- to 2.8-fold, respectively (Fig. 5a, b). *NeoLPAAT1* overexpression dramatically increased TAG content in the microalga.

### Fatty acid composition analysis

**Fig. 6:**
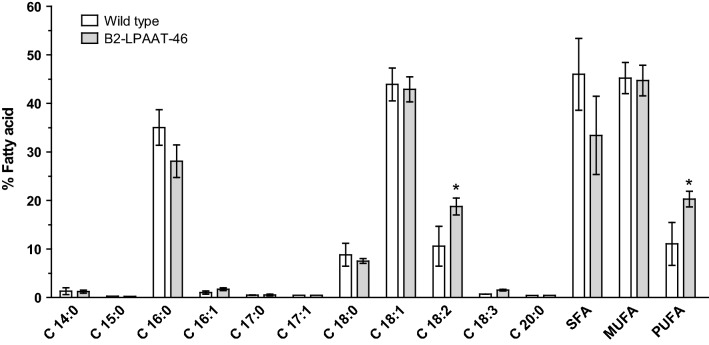
Fatty acid composition in transformant B2-LPAAT-46. FAME of transformant B2-LPAAT-46 and wild type were analyzed using GC–MS. Fatty acid C18:2 and PUFA increased in transformant B2-LPAAT-46 when compared to wild type. SFA, MUFA and PUFA are Saturated, Monounsaturated and Polyunsaturated fatty acids. Each value represents mean ± SD (n = 3). Significant difference between transformant B2-LPAAT-46 and wild type is indicated (*p < 0.05, *t* test)

Because fatty acid (FA) composition can affect the quality and cost of natural edible oils and biofuels, we evaluated whether *NeoLPPAT1* overexpression would have any effect on FA composition. TAG was extracted from transformant B2-LPAAT-46 and wild type under +N condition harvested on day 33 and 38, respectively. Fatty acid methyl esters (FAME) obtained by transesterification of TAG were analyzed using GC–MS (Fig. 6). FAME in the chain-length range of C14–C20 was determined by comparison with the standard reference. The palmitic acid (C16:0) and oleic acid (C18:1) were the most abundant FA in the TAG (Fig. 6). The linoleic acid (C18:2) in transformant B2-PLAAT-46 compared to wild type increased to 19% from 11%; the rest is not significantly different. The overall saturated fatty acid and monounsaturated fatty acid were not significantly different from the wild type, whereas polyunsaturated fatty acid was increased to 20% from 11% (Fig. 6). Thus, a slight alteration of the FA composition in transformant B2-LPAAT-46 was due to overexpression of *NeoLPAAT1*.

### Long‑term stability of transformants

Transformants AR-LPAAT and B2-LPAAT were subcultured (every 2 weeks) in solid BBM over 150 generations in a period of about 6 years. Neutral lipid accumulation in the transformants were periodically checked by Nile red staining; higher lipid-accumulation than wild-type trait was observed in all transformants used in this study, indicating the *NeoLPAAT1* overexpression stability.

## Discussion

This study is based on the overexpression of endogenous plastidial *NeoLPAAT1* in *N. oleoabundans* to increase TAG accumulation for sustainable production of natural edible oils and biofuels. The plastidial LPAAT of *C. reinhardtii* and the putative plastidial LPAAT of other microalgae with sequenced genomes, including *Volvox carteri, Ostreococcus lucimarinus* and *Coccomyxa subellipsoidea*, belongs to the same subcluster as LPAAT1 from cyanobacteria and plants, suggesting a common origin of the plastidal isoform of LPAAT1 [11, 32]. Similar to the well characterized CrLPAAT1 of the model microalga *C. reinhardtii* [11] and AtLPAAT1 of the model plant *Arabidopsis thaliana* [15], plastidial NeoLPAAT1 was found to contain all four canonical motifs attributed to LPAAT proteins, two hypothetical membrane-spanning domains and a putative chloroplast transit peptide (Fig. 1a). NeoLPAAT1 had the closest evolutionary relationship with CvLPAAT1 of Treboxiophycean *C. variabilis* but was quite distantly related to CrLPAAT1 of Chlorophycean *C. reinhardtii* (Fig. 1b). This result is consistent with previous report that diacylglycerol acyltransferase type 2 (NeoDGAT2) of *N. oleoabundans* has the closest evolutionary relationship with CvDGAT2 of *C. variabilis* but is quite distantly related to CrDGAT2A (DGTT4) of *C. reinhardtii* [33]. However, *N. oleoabundans* has been classified based on uninucleate cell morphology to class Chlorophyceae [34]. Classification of *N. oleoabundans* using 18S and 28S rDNA sequence remains to be verified. *N. oleoabundans* possesses ER-located NeoDGAT2 [33] and plastidial NeoLPAAT1 (in this study), suggesting the existence of two distinct and parallel TAG biosynthesis pathways. Similar incidents of duplicated sets of TAG assembly enzymes have been observed in *C. reinhardtii*: CrLPAAT1 and CrDGAT1 located in plastid [11, 35], and CrLPAAT2 and CrDGAT2 located in ER [16, 36].

Among the *NeoLPAAT1*-overexpressing transformants, B2-LPAAT-46 exhibited the highest neutral lipid content which increased to 1.6-fold compared to the maximum content in wild type (Fig. 3b). The lipid content and productivity were dramatically increased under –N condition when compared to +N condition: in transformant B2-LPAAT-46, total lipid content and productivity increased 3.8- and 1.9-fold, respectively, TAG content and productivity increased 4.8- and 2.2-fold, respectively; in wild type, total lipid content and productivity increased 3.9- and 2.5-fold, respectively, TAG content and productivity increased 4.9- and 3.2-fold, respectively (Fig. 5a, b). The results agreed well with previous report that *N. oleoabundans* begins accumulating lipids with the application of minimal N starvation, just following exhaustion of exogenous N. In addition, *N. oleoabundans* exhibits only a small decrease in growth and drastically higher lipid content under N starvation condition; concurrent growth and lipid accumulation result in higher lipid productivity [37]. Lipid productivities of wild type and transformant B2-LPAAT-46 may further increase when the cells cultured under optimal conditions. The lipid productivities of *N. oleoabundans* have been reported with different experimental conditions resulting in large variations in performance, i.e. total lipid productivities vary from 9 to 134 mg/L/day [27, 29, 37, 38] and TAG productivities vary from 6 to 216 mg/L/day [27, 39], making it difficult to compare the reports with each other. The TAG content of 55.40 ± 5.56% DCW produced by the transformant in this study (Fig. 3b) was the highest in comparison to those produced by *LPAAT1*-overexpressing microalgae reported so far [11, 17] and also 20% higher than that (46.1 ± 1.6% DCW) produced by *N. oleoabundans* overexpressing *NeoDGAT2* [27]. The results suggest that the plastidial pathway also plays an important role in TAG biosynthesis in *N. oleoabundans*. The FA composition in transformant B2-LPAAT-46 was slightly altered; the linoleic acid (C18:2) increased to 19% from 11% when compared to wild type (Fig. 6). Whether C18:2-acyl-CoA is a preferred substrate of NeoLPAAT1 remains to be determined.

Silencing or down regulation of the non-required heterologous genes when expressed at high levels has been reported in *C. reinhardtii* [40, 41]. Heterologous gene silencing has also been observed in *N. oleoabundans*; when the transformants introduced with *Gfp* gene [20] continuously maintained in solid BBM medium for over a year, the green fluorescent protein activity seems to diminish [27]. However, overexpression of endogenous *NeoDGAT2* in *N. oleoabundans* has been shown to be stable [27]. Therefore, to avoid heterologous gene silencing, endogenous *NeoLPAAT1* was used in this study. Transformants AR-LPAAT and B2-LPAAT were continuously maintained in solid BBM over 150 generations in a period of about 6 years. All transformants used in this study were periodically checked for neutral lipid accumulation by Nile red staining and found to have higher lipid accumulation than wild-type, indicating the *NeoLPAAT1* overexpression stability.

## Conclusions

We characterized the plastidial NeoLPAAT1 sequence and successfully created *N. oleoabundans* transformant overexpressing NeoLPAAT1 with considerably increased TAG content and productivity. A slightly altered fatty acid composition was detected in the transformant compared to wild type. Stability of *NeoLPAAT1* overexpression was observed in the transformant continuously maintained in solid medium over 150 generations in a period of about 6 years. The considerably increased TAG content and productivity in *N. oleoabundans* by overexpression of *NeoLPAAT1* are important for products derived from microalgal TAG to achieve economic viability. Plastidial LPAAT1 can be a candidate for target genetic manipulation to increase TAG content in other microalgal species with desired characteristics for production of natural edible oils and biofuels.

## Methods

### Strain and growth conditions

*Neochloris oleoabundans* strain UTEX 1185, obtained from the Algal Culture Collection at the University of Texas, was cultured in liquid or in solid (1.5% Difco Bacto agar) Bold’s basal medium (BBM) [42, 43] under constant illumination of 55–60 μmol photons/m^2^/s at 30 °C. Cultures in liquid medium started with cells at density of ~ 1.5 × 10^7^ cells/mL (OD_750_ = 0.3). For cell growth under nitrogen-sufficient ( +N) condition, cultures were maintained in 500 mL Erlenmeyer flasks containing 200 mL of BBM; the flasks were sealed and shaken at 100 rpm. Cell density of the cultures was evaluated using hemocytometer for cell counting and spectrophotometer for OD_750_. Doubling time of the cells was calculated as described [29]. For nitrogen-starvation (−N) condition, the cells grown in BBM at exponential phase were harvested, washed and resuspended in 200 mL of BBM without NaNO_3_ (BBM-N) in 500 mL Erlenmeyer flasks, shaken at 50 rpm and supplied with 50 L/h bubbling-filtered air.

### NeoLPAAT1 sequence analysis

The 1,023-bp *LPAAT* cDNA sequence of *N. oleoabundans* encoding 340 amino acids (GenBank: MF706164; protein id: AUS8446) has been cloned previously as plasmid pYES2-FLPAT34 [21]. We designated this protein as NeoLPAAT1. To further analyze the NeoLPAAT1 sequence, various methods were performed as follows. The NeoLPAAT1 was aligned with other LPAAT1 using ClustalW multiple alignment program [22]. The membrane-spanning domains were predicted by TMHMM v2.0 [24]. The chloroplast transit peptide of the NeoLPAAT1 was predicted using PredAlgo [25]. The phylogenetic tree of NeoLPAAT1 was constructed using the neighbor-joining method in MEGA 7 [26].

### Construction of transformation vectors

To construct *NeoLPAAT1* cDNA under the control of promoters *HSP70*-*RBCS2* (*AR*) [44] and *β2*-*tubulin* (*β2*-*Tub*) [45] of *C. reinhardtii*, plasmids pAR-LPAAT and pB2-LPAAT harboring the gene cassettes *AR*-*NeoLPAAT1*-*3′rbcS2* and (*β2*-*Tub)*-*NeoLPAAT1*-*3′rbcS2*, respectively (Fig. 2a), were constructed by replacing the *AR*-*ChGfp*-*3′rbcS2* fragment of pChGFP-Hyg3 [20] with the PCR fragments containing: (i) *AR* promoter from pCB740 [44] or *β2*-*Tub* promoter from pHyg3 [46] (ii) *NeoLPAAT1* cDNA (GenBank: MF706164) from pYES2-FLPAT34 [21], and (iii) 3′UTR of *3′rbcS2* from pCrGFP [47]. Both pAR-LPAAT and pB2-LPAAT contained hygromycin B resistance gene *Hyg3* [20, 46] used as a selectable marker.

### Transformation of *N. oleoabundans*

To generate transformants overexpressing *NeoLPAAT1*, plasmids pAR-LPAAT and pB2-LPAAT were transformed into the *N. oleoabundans* nuclear genome using electroporation as described previously [20]. *N. oleoabundans* cells were electroporated using a Gene Pulser (Bio-Rad Labs) set electric field strength at 1000 V/cm, capacitance at 25 μF and resistance at 200 Ω. The electroporated cells were spread on a BBM agar plate containing 5 μg/mL hygromycin B. After incubation for 2 weeks, the resulting transformants AR-LPAAT and B2-LPAAT appeared.

### Genomic PCR analysis

The integration of *NeoLPAAT1*-expression cassettes in the nuclear genome of *N. oleoabundans* were verified using genomic PCR. The genomic DNA of *N. oleoabundans* was isolated as described [48]. PCR was performed with primers specific to *NeoLPAAT1* coding sequence: LPAAT-F1 (CGGGATCCTAGCTAGCATGCTGTGCCCCACTGTCG) and LPAAT-R10 (GATGGCAGGGTGAATCACGAGTTTAACTTTGCCTGG). The touchdown PCR was carried out 11 cycles for the first phase (denature at 98 °C for 10 s, annealing at 80 °C for 30 s with a 1 °C decrease in every successive cycle, extension at 72 °C for 30 s) and 25 cycles for second phase (denature at 98 °C for 10 s, annealing at 70 °C for 30 s, extension at 72 °C for 30 s, including initial denaturation at 98 °C for 3 min and final extension at 72 °C for 7 min). The PCR product was investigated in a 1.5% agarose gel and further verified by DNA sequencing analysis. The amplicon of the *NeoLPAAT1* coding sequence was 943 bp.

### Nile red fluorescence assay

To analyze the level of neutral lipids, cells grown under +N condition ( ~ 1.5 × 10^7^ cells/mL) was stained with fluorescent dye Nile red dissolved in acetone to final concentration of 1 μg/mL and incubated in the dark for 10 min. The fluorescence intensity of the stained cells in a 96-well plate was determined using a spectrofluorometer (Beckman Coulter DTX-880, USA) with excitation at 535 nm and emission at 574 nm. Specific fluorescence intensities were obtained by subtracting the autofluorescence of the unstained cells and normalized by cell numbers.

### Quantitative real time PCR analysis

Relative *NeoLPAAT1* transcript abundance was quantified using quantitative real-time PCR (qPCR). Total RNA was extracted from cells cultured under −N condition for 1 day using TRI Solution (GeneMark, Taiwan). The cDNA was prepared from the total RNA with oligo (dT)18 primer using RevertAid H Minus First Strand cDNA Synthesis Kit (Thermo Scientific, Canada). The cDNA was amplified using KAPA SYBR FAST qPCR Kit (Kababiosystems, USA) with *NeoLPAAT1*-gene specific primers: LPAAT-RT-F1 (GAGCTTCCTGGACATTTACACGC) and LPAAT-RT-R1 (CAGCTCGCTGCATTGTTTTAGG); and endogenous *Actin* (*NeoActin*)-gene specific primers: NeoActin-F1 (ACACTGTGCCCATCTATGAGGG) and NeoActin-R1 (CTTGATGTCACGCACGATTTCG). Quantitative RT-PCR analysis was performed using Mastercycler realplex4 and realplex software (Eppendorf, Germany). The *NeoLPAAT1* transcript level was normalized to a reference, *NeoActin* transcript.

### Lipid extraction and quantification

Total lipids of *N. oleoabundans* grown under +N and −N condition were extracted based on modified Bligh and Dyer method [49]. The cell pellet of approximately 80 mg (50 OD_750_) was suspended in chloroform:methanol (1:2, v/v). The cells were lysed using vortexing at 2700 rpm (Vortex Genie2 G560E, Scientific Industries, USA) including 0.5 mm glass beads, then chloroform:water (1:1, v/v) was added to the mixture. Chloroform phase was collected and evaporated using nitrogen gas. Total lipids were determined gravimetrically. The TAG was subsequently separated from total lipids using thin-layer chromatography (TLC) with solvent hexane:diethyl ether:acetic acid (70:30:1, v/v/v) and a reference substance, glyceryl trioleate (92860 Sigma-Aldrich, USA). TAG was quantified using iodine staining and Quantity One 1-D analysis software (Bio-Rad Labs., USA). Total lipid and TAG content was calculated as percentage of dry cell weight (% DCW). DCW of a sample was determined gravimetrically after drying the cells. The lipid productivity was calculated using the formula *P*_Lipid_ (mg/L/day) = [*C*_Lipid_ (mg/mg) × DCW (mg/L)]/Time (day), where *C*_Lipid_ is lipid content of cells, DCW is dry cell weight, and Time is the cultivation period, as described [27, 38].

### Fatty acid composition analysis

To generate fatty acid methyl esters (FAME) by transesterification, TAG extracted from TLC was incubated with 5% (v/v) sulfuric acid in methanol at 70 °C for 3 h in the presence of an internal standard, glyceryl trinonadecanoate (91988 Sigma-Aldrich). FAME analysis was carried out using GC–MS (Agilent 7890A GC system and Agilent 5975C inert XL MSD with Triple-Axis Detector) equipped with Agilent DB-WAX column (30 m length, 0.25 mm i.d., 0.25 μm film thickness). The oven temperature was increased from 50 to 250 °C at a rate of 3 °C/min, the injector and detector temperature were 240 °C and 250 °C, respectively. Helium was used as the carrier gas at flow rate of 1 mL/min. The mass spectra were analyzed using MSD ChemStation software (version E.02.00.493, Agilent) and compared with the NIST08.L database. Fatty acid composition was calculated as percentage of the total fatty acids present in the sample.

### Statistical analysis

The statistical differences between wild type (used as control) and transformant samples were analyzed using two-tailed student’s *t* test of SPSS Base 16.0 software (SPSS, USA).

## Abbreviations

LPAAT: lysophosphatidic acid acyltransferase
AGPAT: 1-acyl-*sn*-glycero-3-phosphate acyltransferase
DGAT: diacylglycerol acyltransferase
DCW: dry cell weight
GC–MS: gas chromatography–mass spectrometry
N: nitrogen
qPCR: quantitative real-time PCR
FA: fatty acids
FAME: fatty acid methyl ester
MUFA: monounsaturated fatty acid
PUFA: polyunsaturated fatty acid
SFA: saturated fatty acid
TAG: triacylglycerol
TLC: thin-layer chromatography
